# The prognostic role of pretreatment thrombocytosis in gastric cancer: A systematic review and meta-analysis

**DOI:** 10.1097/MD.0000000000011763

**Published:** 2018-08-03

**Authors:** Chen Yang, Hui Jiang, Shaozhuo Huang, Hui Hong, Xiaowen Huang, Xiaojie Wang, Weixin Liao, Xueyi Wang, Xuewen Chen, Liming Jiang

**Affiliations:** a The First School of Clinical Medicine; b School of Traditional Chinese Medicine, Southern Medical University, Guangzhou, Guangdong; c Department of Medical Radiology, Zhejiang Cancer Hospital, Hangzhou, Zhejiang, China.

**Keywords:** gastric cancer, meta-analysis, platelet count, prognosis

## Abstract

**Background:**

The association between platelet count and the prognosis of patients with gastric cancer has already been reported by numerous studies. As the reports are inconsistent, we conducted this meta-analysis to evaluate the prognostic significance of platelet count in patients with gastric cancer.

**Methods:**

A comprehensive search was performed in PubMed, Embase, and the Cochrane Library to identify relevant studies, which evaluated the prognostic impact of pretreatment platelet count in patients with gastric cancer. Data was pooled using a random effect model to calculate hazard ratios (HRs) and odds ratios (ORs) with 95% confidence intervals (CIs).

**Results:**

Around 10 studies were included, comprising 8166 patients in total. The result showed that patients with thrombocytosis had significant worse overall survival (HR 1.57, 95% CI 1.36–1.81, *P* < .001) than those with normal platelet count, and were associated with advanced clinical stage (OR, 1.57; 95% CI, 1.15–2.13; *P* = .004), deeper tumor invasion (OR,3.49; 95% CI, 2.48–4.91; *P* < .001), and higher risk of recurrence (OR, 2.28; 95% CI, 1.55–3.35; *P* < .001).

**Conclusion:**

Pretreatment thrombocytosis is a potential effective predictor of overall survival (OS) for patients with gastric cancer and is correlated with higher risks of recurrence, serosal invasion, and advanced stage.

## Abstract 2

Supplemental Digital Content is available in the text

## 1 Introduction

As one of the most common malignant tumors, gastric cancer (GC) is a worldwide leading cause of cancer-related death. It took life from nearly 500,000 people during 2015 in China.^[1]^ At present, the 5-year survival rates of GC patients are under the expectation because patients after surgical resection generally suffer from high risk of local recurrence and distal metastasis. Complications such as thrombosis are important causes of cancer-related deaths as well.^[2]^ Notably, it is universally acknowledged that hemostasis systemic complications occur in GC. Early in 1865, Armand Trousseau first reported that venous thrombosis was prone to form in patients with gastric cancer, and this phenomenon was termed Trousseau's syndrome. Patients with GC show a significant risk of venous thromboembolism (VTE) first year after diagnosis (>5%), while for patients with advanced stages, the risk reaches 12% to 17%. In addition, compared with patients without VTE, patients with VTE had shorter median overall survival.^[3,4]^

Platelets have been well documented to be implicated in tumor progression and thrombosis formation. With multiple ways to promote tumor growth, survival and metastasis, platelets are considered as a “tumor promotor.”^[5–7]^ Studies with various types of cancers, including breast cancer, lung cancer, renal cell carcinoma, gastrointestinal cancer, ovarian or other gynecologic cancers, demonstrated that the elevation of platelet count consistently indicated poor prognosis.^[8–10]^ Furthermore, the interaction between tumor cells and platelets could lead to general hypercoagulability status,^[11]^ correlating with an increased risk of thrombotic complications which are also essential factors for causing poor prognosis.

A number of studies have combined platelet count and other parameters into different indices, such as platelet-lymphocyte ratio (PLR). These indices are generally considered as inflammatory markers evaluating inflammatory status of the body, which are supposed to be better predictors than platelet. However, the results of relevant studies were inconsistent.^[12–14]^ It has been widely acknowledge that lymphocyte is not concerned with thrombus formation. Considering the closer relationship of tumor metastasis with platelet than with lymphocyte, using platelet count alone may be more appropriate to predict tumor metastasis, recurrence, as well as the prognosis of GC patients. Moreover, platelet count measurement is available in ordinary clinical laboratories, making it practical as a prognostic marker. Therefore, we conducted this meta-analysis to evaluate the prognostic role of platelet count in gastric cancer.

## 2 Materials and methods

### 2.1 Literature search

**Figure 1:**
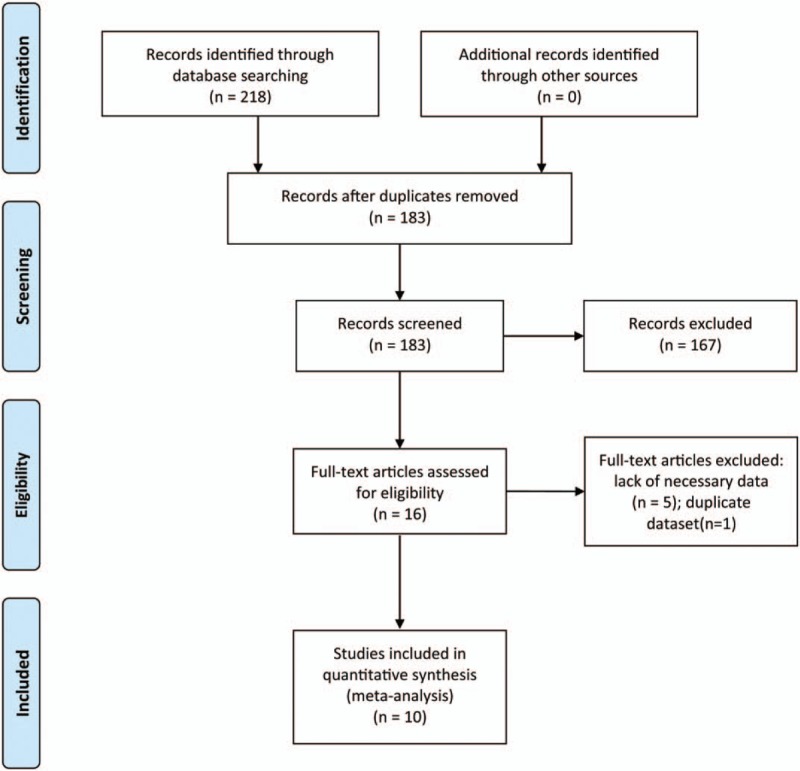
Flow diagram of the literature screening.

This study was carried out according to Preferred Reporting Items for Systematic Reviews and Meta-Analyses (PRISMA) 2009 guidelines.^[15]^ PubMed, Embase, and the Cochrane Library up to June 2017 were systematically searched using the following terms: (“thrombocytosis” OR “thrombocythemia” OR “platelet count”) AND (“gastric cancer” or “gastric adenocarcinoma” or “stomach tumor” or “stomach neoplasms” or “gastric carcinoma”). No language and publication type was restricted. To identify potential relevant studies, the reference lists of all studies were also scanned. All articles were assessed independently by 2 investigators. And questions were resolved by discussion. The selection process of the articles is shown in Figure 1. This study did not require the ethic approval and informed consent due to all analyses were carried out based on the data extracted from previous published trials.

### 2.2 Inclusion and exclusion criteria

The studies were included if they met the following criteria: the diagnosis of gastric cancer was confirmed by pathological examination; platelet count was measured before treatment by a peripheral blood test; providing available data (hazard ratios (HRs) and 95% confidence interval (CI) on overall survival, or the number of patients with or without lymph node metastasis, the depths of tumor invasion or the stage of gastric cancer). If HRs were not directly reported, the estimation of the HR could be calculated from other data^[16]^.

Exclusion criteria were as follows: letters, conference abstracts, review articles, case reports, expert opinions, and posters; studies lacking necessary data; studies referring to any previous treatment before the peripheral blood test; and nonhuman researches. The selection of studies was conducted independently by 2 investigators.

### 2.3 Data extraction

Data was evaluated and extracted independently by 2 investigators, and disagreement or questions were resolved by discussion. The relevant information recorded for each study was listed as follows: study information: first author, year of publication, country, study design, and sample size; patient information: age, treatment strategy, follow-up time, the cut-off value of platelet count, depths of tumor invasion or different stages of gastric cancer, the number of patients with or without recurrence, and the data of HR and 95%CI or other data which can reconstruct HR and 95%CI. The quality of the included studies was assessed according to Newcastle–Ottawa scale (NOS), which was designed for retrospective and prospective studies.^[17]^ Studies scored 6 points and above were defined as high-quality studies.

### 2.4 Statistical analysis

In this meta-analysis, we calculated pooled HRs from each study of their HRs and 95% CIs, which were obtained directly from original articles or were calculated from indirect data on the basis of the methods reported by Tierney et al.^[16]^ Odds ratios (ORs) were calculated to estimate the association between platelet count and clinicopathological characteristics. *I*^*2*^ was used to evaluate the heterogeneity of pooled outcomes. *I*^*2*^ >50% represented the existence of significant heterogeneity among included studies. Both HR and OR were calculated by the random-effects model (DerSimonian–Laird method).^[18]^ To explore the possible source of heterogeneity, subgroup analysis of overall survival was conducted according to geographic distributions, cut-off values, and research styles. Sensitivity analysis was conducted by removing 1 study at a time using the “metaninf” STATA command to confirm the robustness of the outcome of this meta-analysis. Egger's linear regression test and the rank correlation test (Begg's test) were used to assess publication bias.^[19,20]^ All statistical analyses were performed by STATA 12.0 (StataCorp, CollegeStation, TX) and RevMan 5.3.5 (Cochrane Collaboration, Software Update, Oxford, London, UK). *P < *.05 was considered statistically significant.

## 3 Results

### 3.1 Literature search and selected studies

The search strategy found 218 records in PubMed, Embase, and Cochrane. After excluding 35 duplicate articles by computer and scanned each record manually, 183 articles were left. Subsequently, 167 were excluded based on title and abstract review, leaving 16 potential relevant full-text articles. After these full-text articles were carefully reviewed, 6 were excluded because of the lack of necessary data (n = 5) and duplicate dataset (n = 1).

Finally, 10 articles with a total number of 8166 patients were included in this meta-analysis. All the included studies were cohort studies (6 retrospective studies and 4 prospective studies) published between 2002 and 2014.^[9,21–29]^ Five studies reported the association between platelet count and tumor differentiation,^[9,25–27,29]^ and 4 studies evaluated the clinical stage,^[25–28]^ and other 4 studies evaluated the depth of tumor invasion.^[9,25,26,29]^ Four studies directly reported HRs and 95% CIs,^[9,22,23,27]^ and 6 HRs were calculated from other data by indirect method ^[21,24–26,28,29]^. Based on the quality assessment of NOS, all the cohort studies were in moderate quality (1 study scored 8, 3 studies scored 7 and 6 studies scored 6). The basic characteristics of the 10 studies are summarized in Table 1, and the quality assessment of the included studies is presented in Table S1.

**Table 1:**
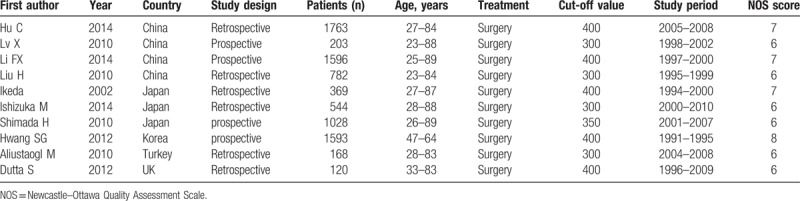
General characteristics of the eligible studies.

### 3.2 Meta-analysis of overall survival

Ten studies involving 8166 patients evaluated the association between platelet count and overall survival (OS) by random-effect model. The pooled analyses showed that patients with elevated pretreatment platelet count had significant poorer OS (HR 1.57, 95% CI 1.36–1.81, *P* < .001) than those with low platelet count (Fig. 1). There was no significant between-study heterogeneity (*I*^*2*^ = 28%, *P* = .18).

Subgroup analyses of OS were conducted according to geographic distribution, study design and cut-off values. The results showed that study design and respective cut-off value did not contribute to the source of heterogeneity (Table 2). But the result of geographic distribution (China vs Japan vs others) might partly explain the source of heterogeneity.

**Table 2:**
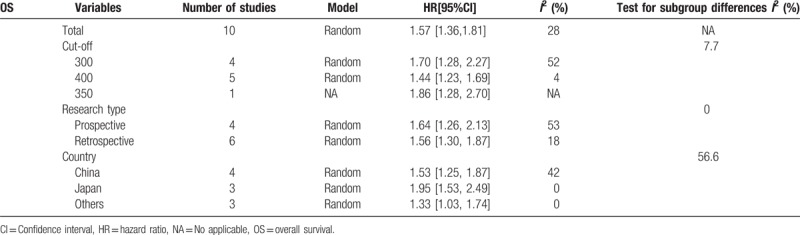
Subgroup analyses results.

As for sensitivity analysis, we removed one study at a time to examine the influence of the individual study to the pooled HRs of OS. The result showed that the pooled HR and its 95% CIs were not affected, and confirmed the robustness of the result of this study (Fig. S1). Egger's linear regression test and the rank correlation test (Begg's test) were conducted to assess the publication bias. Funnel plot shapes did not show an obvious asymmetry (Fig. S2), and the *P* value for Begg's test and Egger's test in platelet count were .592 and .433, respectively. These results demonstrated that no significant publication bias was shown in this meta-analysis.

### 3.3 Associations between platelet count and clinicopathologic parameters

We have extracted and pooled useful data concerning the correlation between platelet count and clinicopathologic parameters from the included articles. The results of tumor differentiation, clinical stage and the depth of tumor invasion provide important guidance for clinicians (Table 3).

**Table 3:**
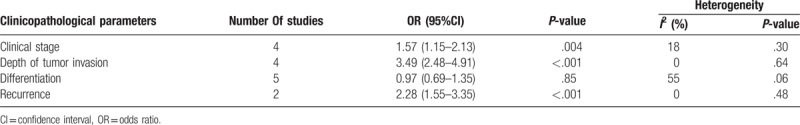
Association between platelet count and clinicopathological parameters.

**Figure 2:**
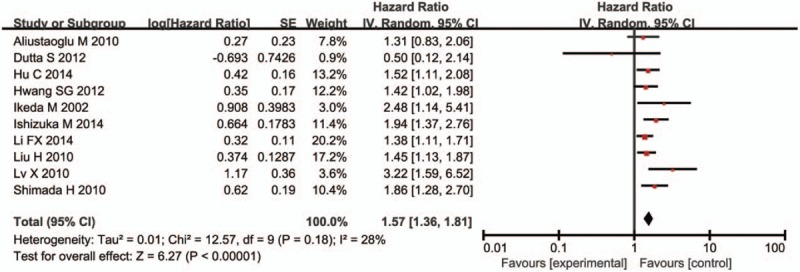
Forest plot of included studies evaluating hazard ratio of platelet count for overall survival.

**Figure 3:**
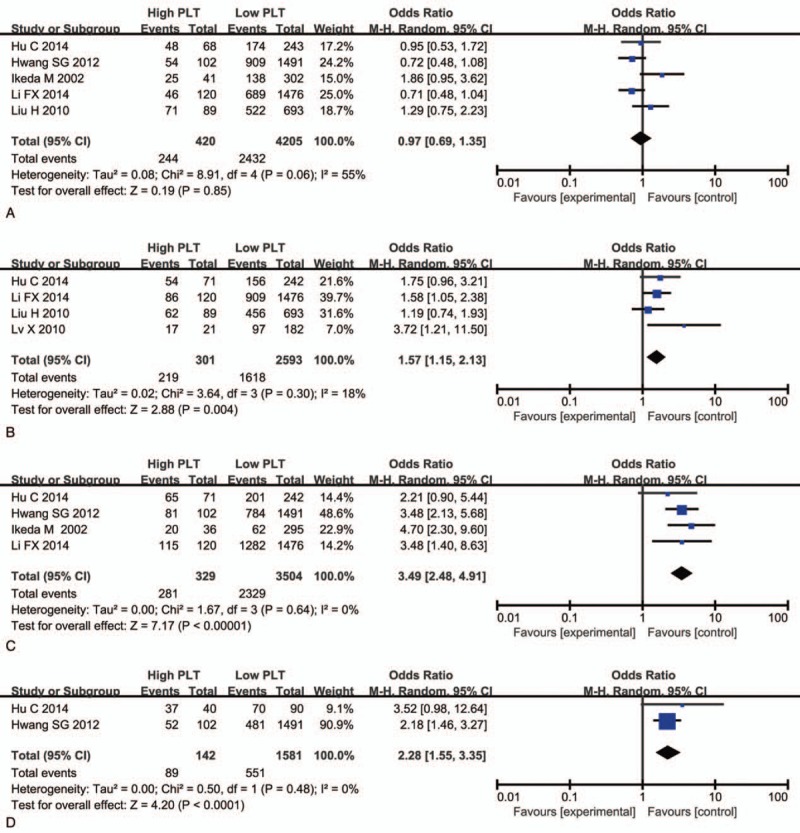
Forest plots of studies evaluating the association between platelet count and tumor differentiation (A), clinical stage (B), depth of tumor invasion (C), and recurrence (D).

Five articles in 10 have reported the association between platelet count and tumor differentiation. In this meta-analysis, we found no significant correlation between elevated platelet count and poor tumor differentiation (OR, 0.97; 95% CI, 0.69–1.35; *P* = .85) (Fig. 2A). As for clinical stage, we extracted data from 4 studies, and found elevated platelet count was associated with advanced clinical stage (OR, 1.57; 95% CI, 1.15–2.13; *P* = .004) (Fig. 2B). With 4 studies providing information between elevated platelet count and the depth of tumor invasion, the result indicated that high platelet count was a significant predicator of deeper tumor invasion (OR, 3.49; 95% CI, 2.48–4.91; *P* < .001) (Fig. 2C). Moreover, the pooled result from 2 studies showed that elevated platelet count was associated with the recurrence of GC (OR, 2.28; 95% CI, 1.55–3.35; *P* < .001) (Fig. 2) (Fig. 3D).

## 4 Discussion

The relationship between thrombocytosis and cancer has been well recognized for a long time. Given that patients with thrombocytosis are at higher risks of tumor metastasis and thrombus formation, it is reasonable to make the conclusion that elevated platelet counts indicates a poorer prognosis.

With multiple ways to facilitate cancer progression, platelets have the ability to promote metastasis, which is one of the most important factors. The prognosis of GC patients with hematogenous metastasis will be significantly worse. Intravasation is the first step as well as a key step in the metastasis of solid tumors. It can be initiated by transforming growth factor-β (TGF-β) released from activated platelets, contributing to the epithelial-mesenchymal transition (EMT) of tumor cells via TGFβ/Smad and NF-κB signaling pathway.^[30]^ Thereafter, platelets are able to protect tumor cells from being damaged by forming cancer embolus when tumor cells are flowing through the blood circulation. Under most circumstances, tumor cells will be attacked by natural killer (NK) cells within the bloodstream because of the low-level expression of major histocompatibility complex (MHC) class I. However, once the cancer embolus is formed, tumor cells will be covered with a coat of platelets. The coat may cause the transference of MHC class I from platelets to tumor cells, resulting in high-level expression of normal MHC class I, and thus protect tumor cells from being attacked by NK cells.^[31,32]^ Moreover, a previous study also found that TGF-β released from platelets can downregulate NKG2D thereby inhibiting NK cells antitumor reactivity, which will further pamper the existence of tumor cells.^[33]^ Apart from NK cells, cytotoxic T lymphocytes (CTLs) also show great importance in tumor immunosurveillance. However, activated platelets can release vascular endothelial growth factor (VEGF) which is able to inhibit the differentiation and development of dendritic cells (DCs), and finally lead to the immune tolerance of CTLs.^[34]^ Further, platelet-derived TGF-β can induce the low-level expression of MHC class II in tumor cells thereby sheltering tumor cells from the cytotoxic effect.^[35]^ Collectively, to form distant metastases, surviving tumor cells also need the assistance of platelets. Platelets can mediate the attachment of tumor cells to endothelial cells and expose subendothelial matrix proteins via adhesion receptors on platelets. The subendothelial matrix proteins can crosslink tumor cells to help establish firm tumor cell arrested within the vasculature. On the other hand, once tumor cells are arrested within the microvasculature of target organs, platelets can release growth factors to promote the growth of tumor cells.^[36]^ In a nutshell, the significance of platelets during the process of tumor metastases is undeniable.

In this meta-analysis, we have demonstrated that elevated platelet count could lead to increased risk of recurrence, advanced stage (III + IV), and serosal invasion (T3 + T4), but would not influence tumor differentiation. These results can be sufficiently explained by the effect of platelets on promoting tumor metastasis. It has also been verified that elevated platelet count was associated with poorer OS in GC patients. Then we used sensitivity analyses of OS by removing one study at a time to assess if individual study influenced the pooled analysis. The result showed that pooled analysis was not obviously influenced by any single study. Considering all the results presented above, it is not hard to tell that pretreatment elevated platelet count is a promising potential predictor of survival for GC patients.

Though another meta-analysis published in 2015 came out with a similar conclusion,^[13]^ it is worth noting that 2 studies adopted in their meta-analysis were excluded in our meta-analysis and 2 additional relevant studies published in 2010 were selected. Moreover, we used clinical stage, tumor differentiation, and recurrence as outcomes and carried out subgroup analyses of the prognostic effect of platelet count on overall survival.

Using thrombocytosis as a predictor is of remarkable clinical significance. Recently, many studies have shown that thrombocytosis was a predictor of the response towards anti-VEGF therapies. A study indicated that metastatic renal cell carcinoma patients with thrombocytosis were more likely to develop drug resistance against anti-VEGF treatment than patients with normal platelet count.^[37]^ Considering the promising prospect anti-VEGF therapies have in gastric cancer treatment, thrombocytosis could be a predictive factor for the efficiency of anti-VEGF therapies in GC patients. Besides, VTE remains as one of the leading causes of cancer-related morbidity and mortality, and thus it is necessary to stratify patients benefiting from anticoagulant thromboprophylaxis. Thrombocytosis has been demonstrated to be related to the high risk of VTE in cancers and thus could be an effective predictor. Patients with thrombocytosis are more likely to undergo antiplatelet therapy. On the other hand, though other systemic inflammatory parameters, such as PLR, neutrophil-lymphocyte ratio (NLR) and C-reactive protein (CRP) were documented to be associated with poor prognosis in cancer patients, they are also found to be limited in several aspects.^[38–42]^ For example, many studies have found that PLR could not act as a significant predictor of OS in various cancers.^[43–45]^ Additionally, though CRP has been identified to be related to the progression of esophageal cancer,^[42]^ the test of CRP is not routinely available in most hospitals. In contrast, platelet count is not only a significant biomarker of OS, but also a measurement of convenience.

Admittedly, our work still has some limitations. Firstly, all the included studies were cohort studies without randomized controlled trials (RCTs). Secondly, only 4 studies directly reported HRs and 95% CIs, the rest of HRs were calculated from related data by the indirect method. Lastly, though the result of subgroup analysis showed that the prognostic value of platelet count was not significantly affected by the discordance of platelet count cut-off, we still have the reason to believe that it created the greatest limitation and deviation in our work. In the future, further meta-analyses including additional studies are needed to verify the accuracy of the results.

In conclusion, pretreatment thrombocytosis is a useful predictor of OS in GC patients and is correlated with higher risks of recurrence, serosal invasion and advanced stage in GC. It can be applied in clinical treatment and can predict treatment outcomes.

## Acknowledgments

We appreciate the work of editors and anonymous reviewers.

## Author contributions

**Conceptualization:** Hui Jiang, Chen Yang.

**Data curation:** Hui Jiang, Chen Yang.

**Formal analysis:** Hui Jiang, Chen Yang.

**Funding acquisition:** Hui Jiang, Chen Yang.

**Investigation:** Hui Jiang, Chen Yang.

**Methodology:** Hui Jiang, Chen Yang.

**Project administration:** Hui Jiang, Chen Yang.

**Resources:** Hui Jiang, Chen Yang.

**Software:** Hui Jiang, Chen Yang.

**Supervision:** Hui Jiang, Chen Yang.

**Validation:** Hui Jiang, Chen Yang.

**Visualization:** Hui Jiang, Chen Yang.

**Writing – original draft:** Hui Jiang, Chen Yang.

**Writing – review & editing:** Hui Jiang, Chen Yang, Shaozhuo Huang, Hui Hong, Xiaowen Huang, Xiaojie Wang, Weixin Liao, Xueyi Wang, Xuewen Chen, Liming Jiang.

## Supplementary Material

Supplemental Digital Content
