# Supplementary material for: The prognostic role of pretreatment thrombocytosis in gastric cancer: A systematic review and meta-analysis
Source: Medicine (Baltimore). 2018 Aug 3;97(31):e11763. doi: 10.1097/MD.0000000000011763 (PMC6081180; doi:10.1097/MD.0000000000011763)

**Supporting information**

Table S1. Details of literature search in the databases.

Table S2. Results of quality assessment

Figure S1. Sensitivity analysis for studies evaluating hazard ratio of platelet count for overall survival.

Figure S2. Funnel plot for studies evaluating hazard ratio of platelet count for overall survival.

PubMed

1.Prognostic Factor

2. Factors，prognostic

3.Prognostic Factors

4.Prognoses

5.Prognosis[Mesh]

6.1 OR 2 OR 3 OR 4 OR 5

7.Platelet counts

8.Thrombocytosis

9.Thrombocythemia

10.7 OR 8 OR 9

11. Gastric Cancer, Familial Diffuse

12. Cancer of the Stomach

13. Cancers, Stomach

14. Cancer, Stomach

15. Stomach Cancer

16. Gastric Cancers

17. Cancer, Gastric

18. Cancers, Gastric

19. Gastric Cancer

20. Stomach Cancers

21. Cancer of Stomach

22. Neoplasms, Gastric

23. Stomach Neoplasm

24. Neoplasm, Stomach

25. Gastric Neoplasms

26. Neoplasm, Gastric

27. Gastric Neoplasms

28. Gastric Neoplasm

29. "Stomach Neoplasms"[Mesh]

30.11 OR 12 OR 13 OR 14 OR 15 OR 16 OR 17 OR 18 OR 19 OR 20 OR 21 OR 22 OR 23 OR 24 OR 25 OR 26 OR 27 OR 28 OR 29

31.6 AND 10AND 30

EMbase

#1 ‘Prognostic Factor’

#2 ‘Factors，prognostic’

#3 ‘Prognostic Factors’

#4 ‘Prognoses’

#5 #1 OR #2 OR #3 OR #4

#6 ‘Platelet counts’

#7 ‘Thrombocytosis’

#8 ‘Thrombocythemia’

#9 #6 OR #7 OR #8

#10 ‘Gastric Cancer, Familial Diffuse’

#11 ‘Cancer of the Stomach’

#12 ‘Cancers, Stomach’

#13 ‘Cancer, Stomach’

#14 ‘Stomach Cancer’

#15 ‘Gastric Cancers’

#16 ‘Cancer, Gastric’

#17 ‘Cancers, Gastric’

#18 ‘Gastric Cancer’

#19 ‘Stomach Cancers’

#20 ‘Cancer of Stomach’

#21 ‘Neoplasms, Gastric’

#22 ‘Stomach Neoplasm’

#23 ‘Neoplasm, Stomach’

#24 ‘Gastric Neoplasms’

#25 ‘Neoplasm, Gastric’

#26 ‘Gastric Neoplasms’

#27 ‘Gastric Neoplasm’

#28 #11 OR #12 OR #13 OR #14 OR #15 OR #16 OR #17 OR #18 OR #19 OR #20 OR #21 OR #22 OR #23 OR #24 OR #25 OR #26 OR #27

#29 #5 AND #9 AND #28

The Cochrane Library

#1 Prognoses or prognostic factor or factors，prognostic or prognostic factors (Word variations have been searched)

#2 Platelet counts or thrombocytosis or thrombocythemia (Word variations have been searched)

#3 Gastric Cancer, Familial Diffuse or Cancer of the Stomach or Cancers, Stomach or Cancer, Stomach or Stomach Cancer (Word variations have been searched)

#4Gastric Cancers or Cancer, Gastric or Cancers, Gastric or Gastric Cancer or Stomach Cancers (Word variations have been searched)

#5Cancer of Stomach or Neoplasms, Gastric or Stomach Neoplasm or Neoplasm, Stomach or Gastric Neoplasms (Word variations have been searched)

#6Neoplasm, Gastric or Gastric Neoplasms or Gastric Neoplasm (Word variations have been searched)

#7MeSH descriptor: [Prognosis] explode all trees

#8MeSH descriptor: [Stomach Neoplasms] explode all trees

#9 #1 or #7

#10 #3 or #4 or #5 or #6 or #7

#11#2 and #9 and #10

| Table S2 Results of quality assessment by Newcstle-Ottawa Scale | | | | | | | | | | | |
| --- | --- | --- | --- | --- | --- | --- | --- | --- | --- | --- | --- |
| First Author | Year | Selection | | | | Comparability | | Exposure | | |  |
|  |  | 1 | 2 | 3 | 4 | 5A | 5B | 6 | 7 | 8 | scores |
| Hu C | 2014 | √ | √ | √ | √ | √ | × | × | √ | √ | 7 |
| Lv X | 2010 | √ | √ | √ | √ | × | × | × | √ | √ | 6 |
| Li FX | 2014 | √ | √ | √ | √ | × | × | √ | √ | √ | 7 |
| Liu H | 2010 | √ | √ | √ | √ | × | × | × | √ | √ | 6 |
| Ikeda | 2002 | √ | √ | √ | √ | × | × | √ | √ | √ | 7 |
| Ishizuka M | 2014 | √ | √ | √ | √ | × | × | × | √ | √ | 6 |
| Shimada H | 2010 | √ | √ | √ | √ | × | × | × | √ | √ | 6 |
| Hwang SG | 2012 | √ | √ | √ | √ | √ | √ | × | √ | √ | 8 |
| Aliustaoglu M | 2010 | √ | √ | √ | √ | × | × | × | √ | √ | 6 |
| Dutta S | 2012 | √ | √ | √ | √ | × | × | × | √ | √ | 6 |

1 indicates exposed cohort truly representative of population; 2, community controls; 3, ascertainment of exposure; 4, outcome of interest not present at start; 5A, cohorts comparable on age and gender; 5B, cohorts comparable on additional factor(s); 6 quality of outcome assessment; 7 follow up long enough for outcomes to occur; 8, complete accounting for cohorts.


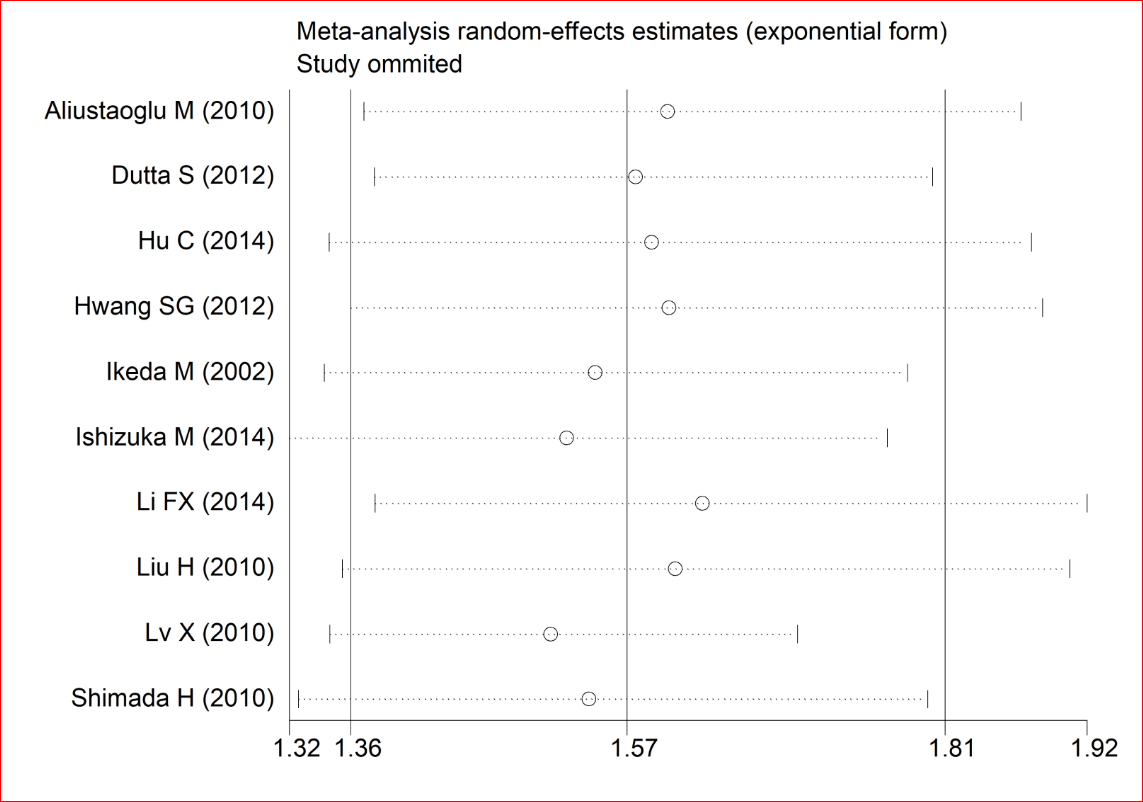


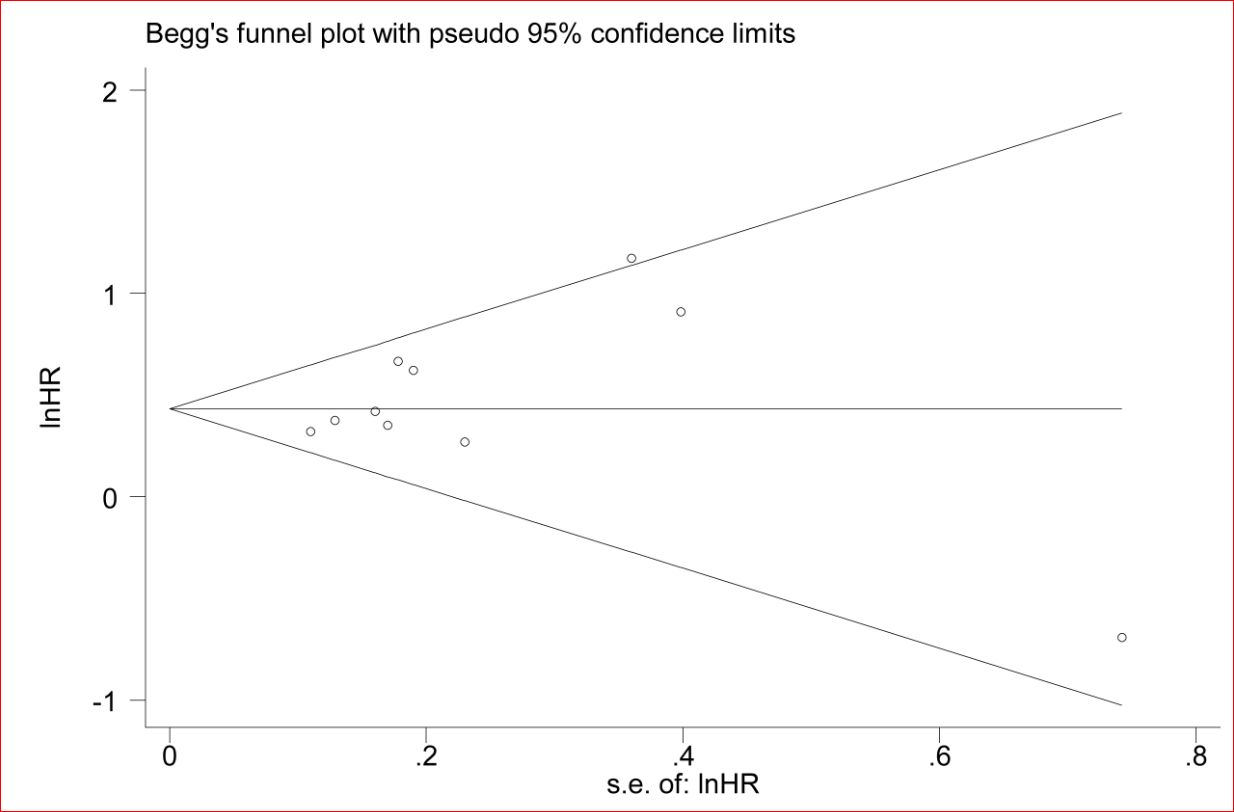

Supplement: Supplemental Digital Content [file medi-97-e11763-s001.doc]
